# Laparoscopic Cholecystectomy Completely Guided by Indocyanine Green Fluorescence: A Case Report and Narrative Review of the Literature

**DOI:** 10.7759/cureus.80561

**Published:** 2025-03-14

**Authors:** Kostas Tepelenis, George Mpourazanis, Demetrios Hadjis, Panagiotis Tsirkas, Konstantinos Lantavos, Konstantinos Karakasis, Apostolos Ntanasis, Elisavet Melissi, Demosthenes E Ziogas, Maria Alexandra Kefala

**Affiliations:** 1 Department of General Surgery, General Hospital of Ioannina G. Hatzikosta, Ioannina, GRC; 2 Department of Obstetrics and Gynecology, General Hospital of Ioannina G. Hatzikosta, Ioannina, GRC; 3 Vascular Unit, Department of General Surgery, University Hospital of Ioannina, Ioannina, GRC; 4 Department of Anesthesiology, General Hospital of Ioannina G. Hatzikosta, Ioannina, GRC; 5 Department of Pediatrics, General Hospital of Ioannina G. Hatzikosta, Ioannina, GRC

**Keywords:** critical view of safety, indocyanine green, intraoperative cholangiography, laparoscopic cholecystectomy, near-infrared imaging

## Abstract

Although laparoscopic cholecystectomy is the cornerstone of treatment for benign gallbladder diseases, the Achilles heel of this operation is the possibility of bile duct injury, which is a rare but potential devastating complication. In recent years, using indocyanine green (ICG) to guide laparoscopic cholecystectomy has gained popularity. It is an innovative method that facilitates the identification of the extrahepatic biliary tract and, occasionally, the cystic artery. Laparoscopic cholecystectomy entirely guided by ICG has been described rarely in the literature. Herein, we described the case of a 48-year-old male who underwent laparoscopic cholecystectomy under complete ICG guidance. A total of 2.5 ml of ICG was administered 45 minutes before the procedure. The extrahepatic biliary tract was visualized using ICG cholangiography. The cystic artery was visualized just 60 seconds after an intraoperative injection of 3 ml of the ICG solution via ICG angiography. Following this, the cystic duct and cystic artery were clipped and divided, and the gallbladder was detached from the liver bed under fluorescence guidance. The total operative time was 43 minutes, with minimal intraoperative blood loss of 10 ml. This technique may provide a safer alternative compared to conventional laparoscopic cholecystectomy under ICG guidance, as the entire procedure is performed under ICG visualization. Nonetheless, the safety and efficacy of this approach should be further evaluated in future case series.

## Introduction

Dr. Eric Muhe reported the first laparoscopic cholecystectomy (LC) in 1985 [1]. Today, it is one of the most common laparoscopic procedures worldwide and has become the treatment of choice for benign gallbladder diseases [2]. However, a significant concern with this procedure is the risk of common bile duct (CBD) injury, which has an estimated incidence of 0.5-2% [3]. The Critical View of Safety (CVS) is considered the safest method for performing LC. Nonetheless, due to complex anatomical variations or pathological conditions, achieving this view may not always be possible [4]. Various alternative techniques exist for such cases, each with its drawbacks.

Near-infrared fluorescence (NIF) imaging, which involves the intravenous administration of indocyanine green (ICG), is an innovative method that aids in identifying the cystic duct (CD), CBD, common hepatic duct (CHD), and the CD-CBD junction through ICG-cholangiography, as well as the cystic artery (CA) via ICG-angiography using fluorescence imaging systems [5]. ICG is a sterile, anionic, water-soluble substance with a relatively hydrophobic characteristic and a molecular weight of 776 Daltons. After being administered intravenously, ICG quickly binds to plasma proteins, is extracted by the liver without modifications, and is nearly exclusively excreted into the bile. When exposed to near-infrared light (700 to 900 nm), ICG absorbs it at a peak of 807 nm and emits fluorescence at a peak of 822 nm, which illuminates vascular and biliary structures. Specialized scopes and cameras can detect fluorescence emission by ICG [6]. In this report, we discuss the case of a 48-year-old male patient who underwent an LC completely guided by ICG fluorescence without switching to the white light mode.

## Case presentation

The patient was a 48-year-old male with no prior history of abdominal surgery. He had experienced three episodes of acute cholecystitis over the previous two years, with the most recent episode occurring three months ago. For the procedure, 25 mg of ICG (brand name Verdye) was diluted in 10 ml of sterile water. We administered 2.5 ml of this solution 45 minutes prior to surgery, while the remaining solution was wrapped in foil for intraoperative use.

**Figure 1:**
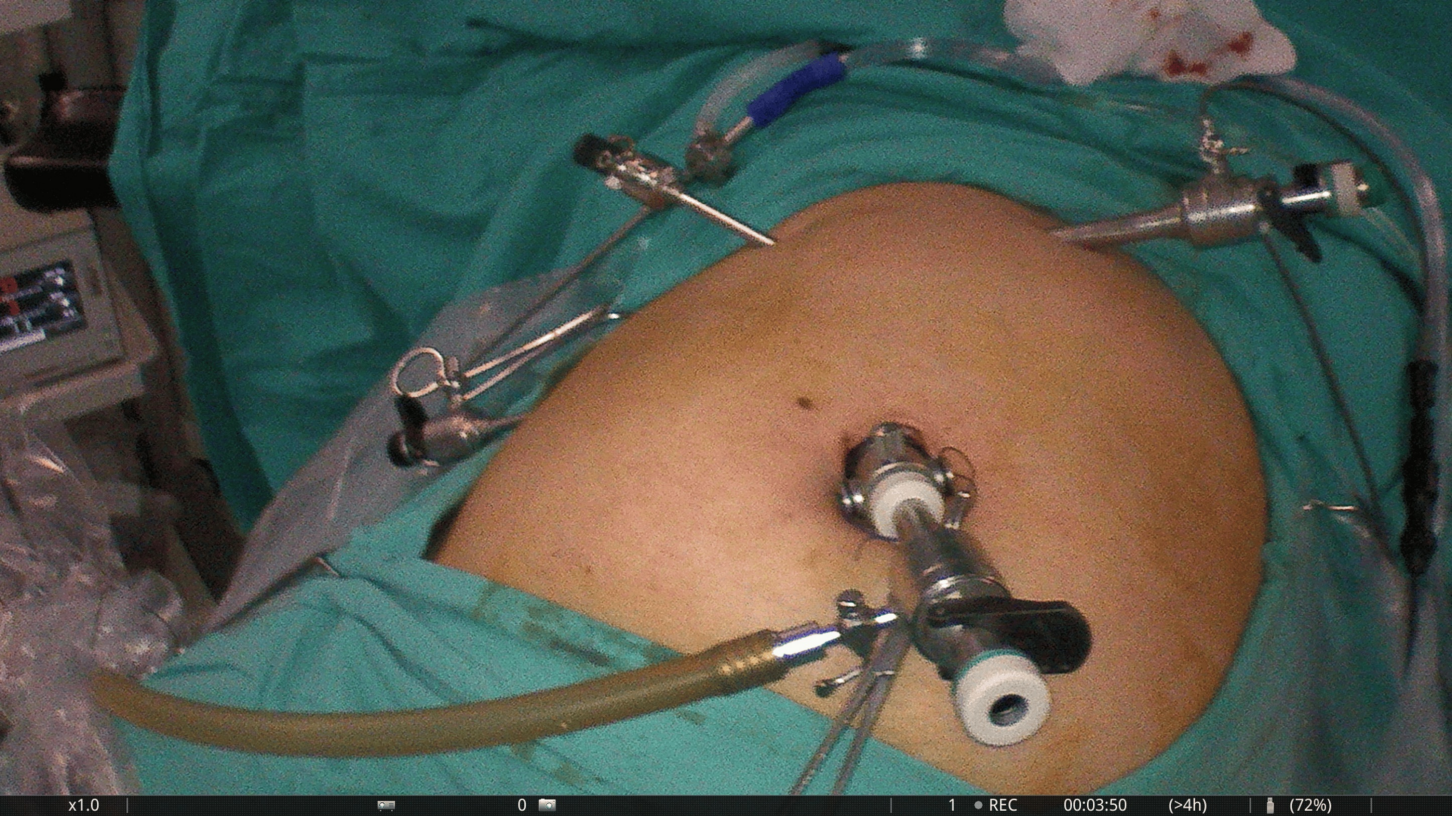
Port placement for laparoscopic cholecystectomy.

**Figure 2:**
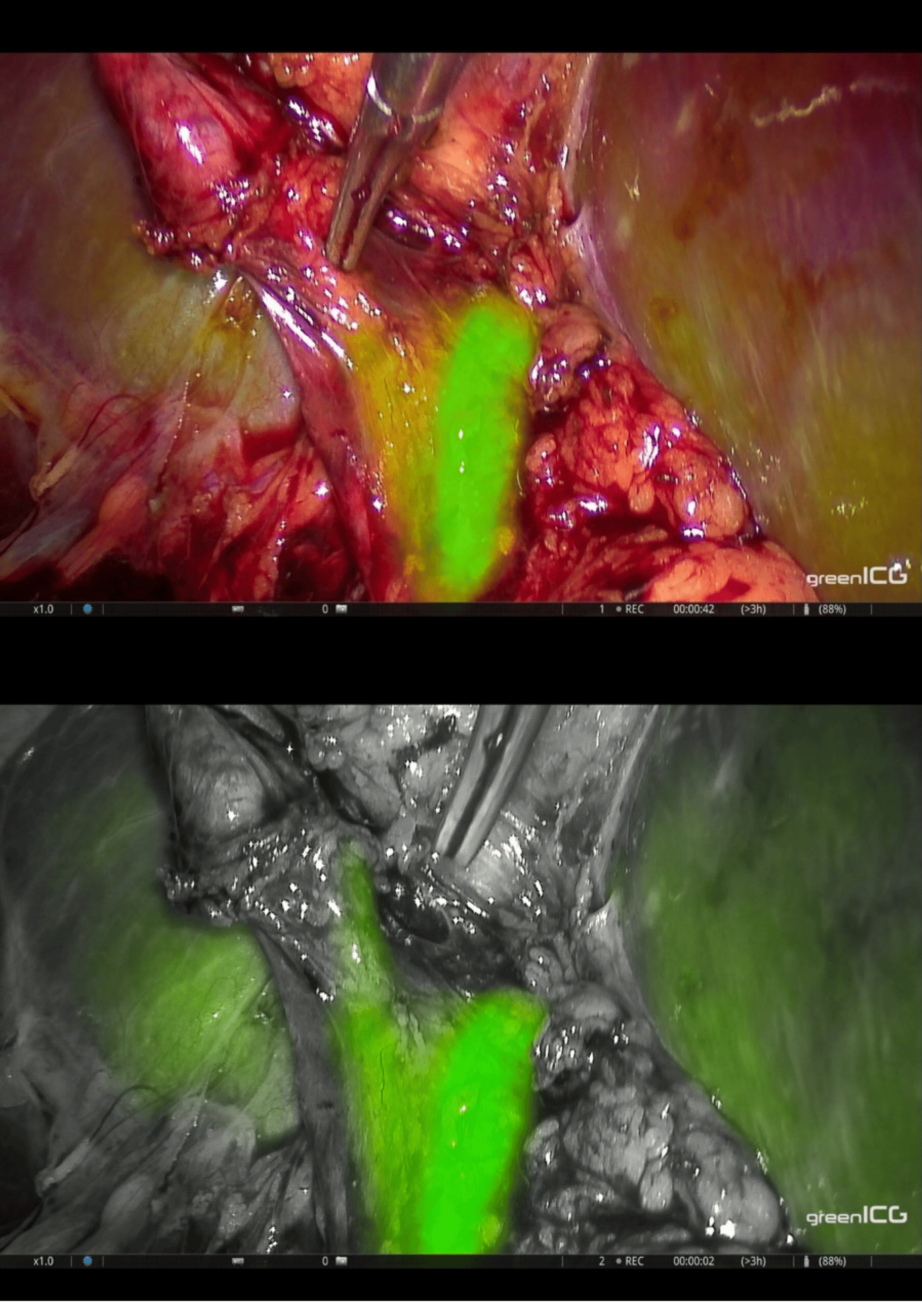
Visualization of the extrahepatic biliary tract through ICG cholangiography. ICG: Indocyanine green

The pneumoperitoneum was established using the open-entry technique (Hasson’s technique). The surgical procedure involved a four-trocar approach and was conducted entirely under fluorescence guidance (Figure 1). Adhesions between the gallbladder and the liver were carefully dissected using monopolar energy. We then incised the peritoneum along the edges of the gallbladder on both sides to open the hepatocystic triangle. This maneuver allowed for the illustration of the extrahepatic biliary tract through ICG cholangiography, facilitating the dissection of the cystic duct (Figure 2).

**Figure 3:**
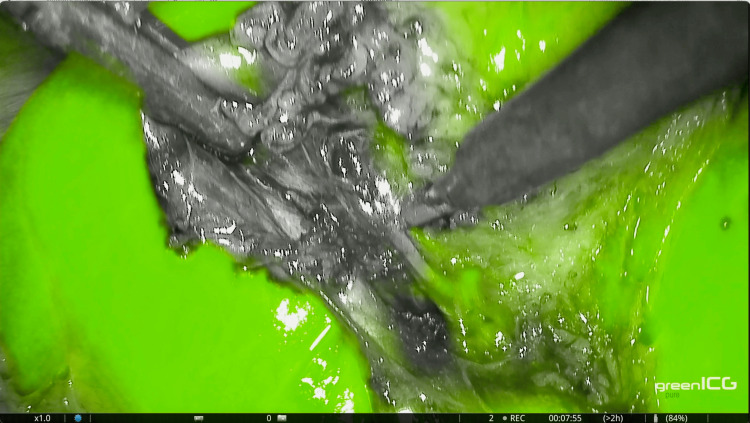
Visualization of the cystic artery through ICG angiography. ICG: Indocyanine green

**Figure 4:**
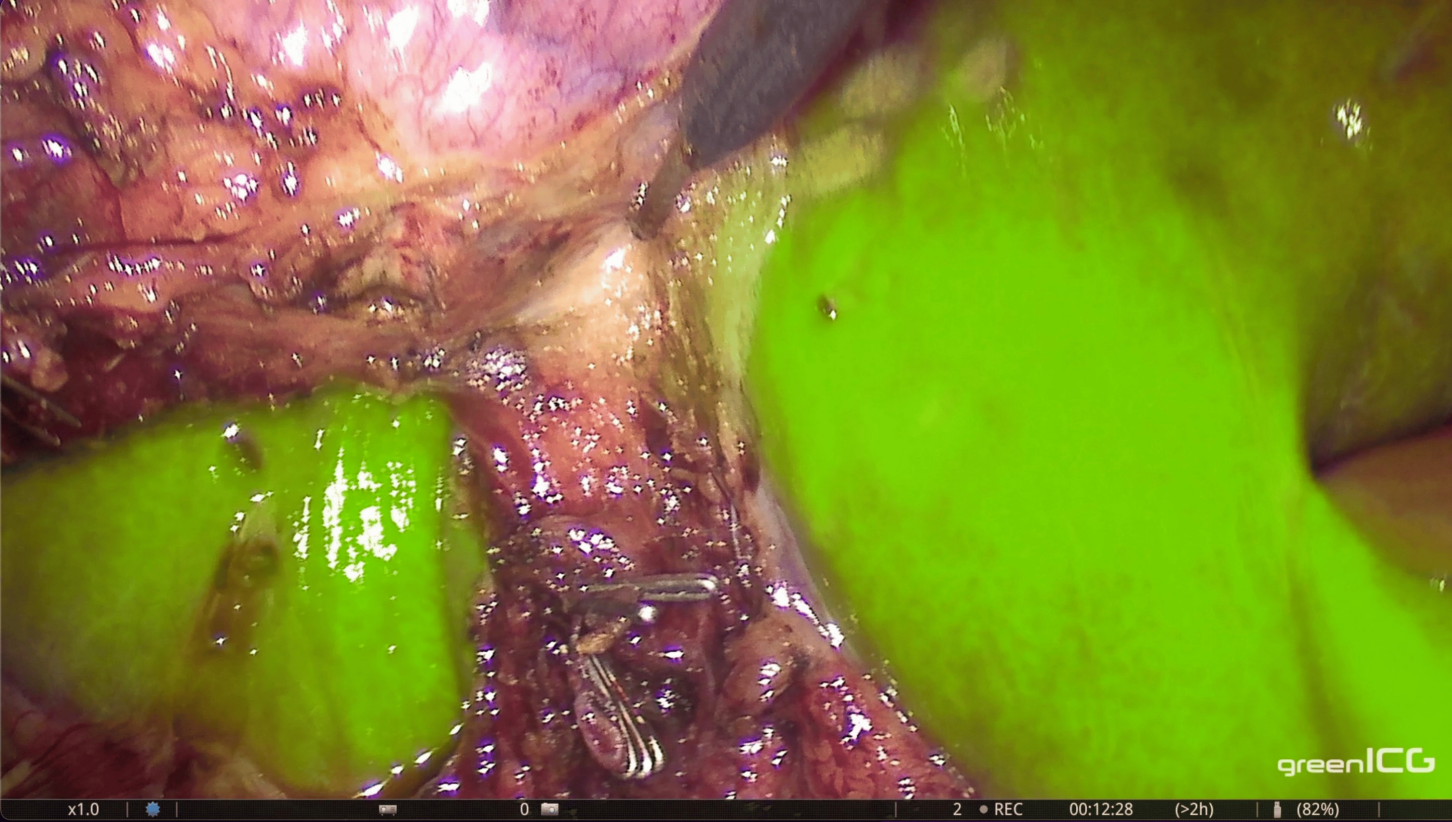
Detachment of the gallbladder from the liver bed under ICG guidance. ICG: Indocyanine green

Following this, we administered 3 ml of the ICG solution along with a 10 ml saline flush intraoperatively. Visualization of the CA occurred 60 seconds after injection and lasted for 35 seconds via ICG angiography (Figure 3). We then continued with the dissection of the cystic artery and subsequently clipped and divided both the cystic duct and cystic artery. The gallbladder was carefully detached from the liver bed under fluorescence guidance (Figure 4). A drain was placed in Morrison’s pouch, and the specimen was extracted using a laparoscopic bag. The operation lasted 43 minutes, and the estimated blood loss was 10 ml. The postoperative period was uneventful. The drain was removed the following morning, and the patient was discharged on the first postoperative day.

## Discussion

Dr. Erich Muhe performed the first LC in 1985 [1]. Today, this procedure is one of the most performed laparoscopic surgeries globally and has become the preferred treatment for benign gallbladder lesions [2]. However, a notable risk associated with the procedure is injury to the CBD, which occurs in approximately 0.5% and 2% of cases [3]. A survey indicates that up to 50% of surgeons may experience CBD injury at some point in their careers [7].

The CVS is regarded as the safest method for performing LC. Nassar et al. conducted a prospective study involving 1,060 consecutive LC to assess the challenges related to CVS and the feasibility of displaying it during each procedure. The operative difficulty was graded according to the Modified Nassar Scale. The authors indicated that CVS could not be achieved in 16.6% of LC. Specifically, CVS was not achieved in 14.4% of cases rated as difficulty level III, 55.6% of cases rated as difficulty level IV, and 92.3% of cases rated as difficulty level V [4]. Alternative methods, such as conversion to open cholecystectomy, retrograde fundus-first laparoscopic cholecystectomy, laparoscopic subtotal cholecystectomy, and intraoperative cholangiography (IOC), each have their drawbacks.

Ishizawa et al. performed the first fluorescent cholangiography using ICG for LC in 2009 [8]. This novel method, known as NIF imaging, involves the intravenous administration of ICG to facilitate the identification of CD, CBD, CHD, and CD-CBD junction through ICG cholangiography, as well as CA via ICG angiography using fluorescence imaging systems [5]. ICG is a sterile, anionic, water-soluble compound with relatively hydrophobic properties and a molecular mass of 776 Daltons. After intravenous administration, ICG binds to plasma proteins, is metabolized exclusively by the liver, and is ultimately excreted into the bile. The peak concentration of ICG in bile is observed 30 minutes to 2 hours after injection, while the peak arterial concentration occurs 1 to 2 minutes post-injection. A specialized camera system emits near-infrared light (700-900 nm), which excites the ICG molecules in the patient, causing the ICG to emit fluorescence. The absorption peak of ICG occurs at 807 nm, and the emission peak is observed at 822 nm. The camera filters are designed to detect the fluorescent light emitted by the ICG, and the images are displayed on an imaging system monitor [6, 9, 10].

According to the International Society for Fluorescence Guided Surgery, for effective illustration of the extrahepatic biliary tract using ICG cholangiography, a dose of 0.05 mg/kg body weight or 2.5 ml should be administered intravenously at least 45 minutes before the procedure. Visualization of the extrahepatic bile ducts should occur pre and post-dissection of Calot’s triangle. For visualization of the CA through ICG angiography, 3-3.5 ml of ICG along with a 10 cc saline flush should be administered intraoperatively, with the CA being detectable 30 to 60 seconds after injection.

In our case report, the patient underwent an LC that was entirely guided by ICG fluorescence. We were able to visualize the CD, CBD, CD-CBD junction, and CHD using ICG cholangiography. Furthermore, the identification of the CA was achieved 60 seconds after the intraoperative administration of ICG, with visibility lasting for 35 seconds, as observed through ICG angiography. The gallbladder was carefully detached from the liver bed under ICG guidance, and the entire procedure was performed without switching to white light mode. Accessory bile ducts and anatomical variants of the right posterior bile duct are found in 21% of cases, which can be recognized with this method [11]. However, none of these situations were observed in our patient. This technique has been previously described in the literature. Takahashi et al. reported an LC that was entirely guided by indocyanine green fluorescence in a patient with gallstones [12]. Future case series should evaluate the benefits and drawbacks of this technique.

Several studies have examined the safety and effectiveness of ICG-guided LC. A meta-analysis conducted by Liu et al. in 2020, which included 11 studies and a total of 2,221 patients, found that the ICG group experienced statistically significant benefits, including a shorter operative time, reduced biliary anatomy identification time, lower blood loss, increased success rates for biliary tract imaging, decreased rates of conversion to open surgery, shorter hospital stays, and lower costs for biliary tract imaging [2]. In 2021, Dip et al. performed a meta-analysis of 16 studies involving 6,673 patients and revealed that ICG cholangiography sizably reduced the rates of CBD injury and conversion to open surgery compared to LC alone [13]. A randomized controlled trial (RCT) by Dip et al. in 2019, which included 639 patients, displayed that ICG cholangiography greatly improved the visualization of the extrahepatic bile duct anatomy compared to LC alone [10]. Another RCT conducted by van den Bos et al. in 2023 with 294 patients demonstrated that ICG-guided LC resulted in earlier identification of relevant extrahepatic biliary anatomy and quicker achievement of CVS [5].

Three studies compared ICG cholangiography to IOC, all reporting equal visualization rates of the extrahepatic bile ducts for both techniques [1, 9, 14]. A meta-analysis of 19 studies involving 772 patients conducted by Vlek et al. in 2019 revealed similar visualization rates of the extrahepatic biliary tract for both techniques [9]. Furthermore, in a meta-analysis of seven studies with 481 patients performed by Lim et al. in 2021, no difference was observed in the illustration of the extrahepatic biliary tract when using ICG cholangiography compared to IOC. Nonetheless, ICG cholangiography did show higher visualization rates for the CHD compared to IOC [1]. Lastly, an RCT involving 120 patients by Lehrskov et al. in 2020 confirmed that ICG cholangiography was non-inferior to IOC in visualizing the extrahepatic bile ducts during LC [14].

The advantages of ICG include its less invasive nature, as it does not require incisions in the CD or CBD, and it does not expose the patient to radiation. Additionally, ICG can help identify the biliary tract before dissection of Calot’s triangle. However, ICG cholangiography requires specialized imaging technology, is costly, and may not be widely available. Another limitation of ICG cholangiography is its tissue penetration capability, which is limited to 5-10 mm. As a result, it may not effectively visualize deeply located bile ducts during LC. This limitation explains the lower visualization rate for CHD compared to the more superficially located CD and CBD, as noted by several authors [1, 5, 9, 15].

In 2023, the European Association for Endoscopic Surgery (EAES) initiated a consensus development conference on ICG fluorescence-guided surgery to establish evidence-based statements and recommendations for the surgical community. ICG cholangiography during LC enhances the identification of extrahepatic biliary anatomy before and after the dissection of Calot’s triangle and may reduce operative time and conversion rates compared to intraoperative imaging. Therefore, ICG cholangiography is recommended, whenever available, to improve the visualization of biliary structures (Grade of recommendation: Strong) [16].

## Conclusions

ICG-guided LC is a straightforward, safe, and feasible technique that provides real-time visualization of the extrahepatic bile ducts and CA. While CVS is generally regarded as the safest method for performing LC, there are situations where it may not be possible. ICG is not designed to replace CVS; rather, it serves as a valuable aid in complex LC cases where CVS cannot be utilized. Performing LC with complete fluorescence guidance may enhance the safety of the surgery. However, verification through a case series will be necessary.
